# Developing a manufacturing process to deliver a cost effective and stable liquid human rotavirus vaccine

**DOI:** 10.1016/j.vaccine.2021.03.033

**Published:** 2021-04-08

**Authors:** Ahd Hamidi, Femke Hoeksema, Pim Velthof, Angelique Lemckert, Gert Gillissen, Alfred Luitjens, Julie E. Bines, Swathi R. Pullagurla, Prashant Kumar, David B. Volkin, Sangeeta B. Joshi, Menzo Havenga, Wilfried A.M. Bakker, Christopher Yallop

**Affiliations:** a Batavia Biosciences BV, Zernikedreef 16, 2333CL Leiden, the Netherlands; b Murdoch Children’s Research Institute, Department of Paediatrics, University of Melbourne, Department of Gastroenterology and Clinical Nutrition, Royal Children’s Hospital, Parkville, Victoria 3052, Australia; c Department of Pharmaceutical Chemistry, Vaccine Analytics and Formulation Center, University of Kansas, Lawrence, KS 66047, USA

**Keywords:** Oral rotavirus vaccine, Fixed bed bioreactor, Manufacturing process, Cost-of-goods modelling, Stable liquid formulation, BSA, Bovine Serum Albumin, CCID50, Cell Culture Infectious Dose 50%, CDAI, Cell Density at Infection, COGs, Cost of Goods, DOE, Design of Experiments, DSP, Down-Stream Processing, DP, Drug Product, DS, Drug Substance, ELISA, Enzyme-Linked Immuno-Sorbent Assay, FFA, Focus Forming assay, FFU, Focus Forming Units in Focus Forming assay, GAVI, Global Alliance for Vaccines and Immunization, HBGA, Histo-Blood Group Antigen, HP-SEC, High Performance Size-Exclusion Chromatography, ICH, International Council for Harmonisation of Technical Requirements for Pharmaceuticals for Human Use, IU, International Unit, LIC, Low Income Countries, LLOQ, Lower Level Of Quantification, MOI, Multiplicity of Infection, MVS, Master Virus Seed, ORV, Oral Rotavirus Vaccine, qPCR, Quantitative Polymerase Chain Reaction, UNICEF, United Nations Children's Fund, USD, United States Dollar, USP, Up-Stream Processing, WHO, World Health Organization

## Abstract

Despite solid evidence of the success of rotavirus vaccines in saving children from fatal gastroenteritis, more than 82 million infants worldwide still lack access to a rotavirus vaccine. The main barriers to global rotavirus vaccine coverage include cost, manufacturing capacity and suboptimal efficacy in low- and lower-middle income countries. One vaccine candidate with the potential to address the latter is based on the novel, naturally attenuated RV3 strain of rotavirus, RV3-BB vaccine administered in a birth dose strategy had a vaccine efficacy against severe rotavirus gastroenteritis of 94% at 12 months of age in infants in Indonesia. To further develop this vaccine candidate, a well-documented and low-cost manufacturing process is required. A target fully loaded cost of goods (COGs) of ≤$3.50 per course of three doses was set based on predicted market requirements. COGs modelling was leveraged to develop a process using Vero cells in cell factories reaching high titers, reducing or replacing expensive reagents and shortening process time to maximise output. Stable candidate liquid formulations were developed allowing two-year storage at 2–8 °C. In addition, the formulation potentially renders needless the pretreatment of vaccinees with antacid to ensure adequate gastric acid neutralization for routine oral vaccination. As a result, the formulation allows small volume dosing and reduction of supply chain costs. A dose ranging study is currently underway in Malawi that will inform the final clinical dose required. At a clinical dose of ≤6.3 log_10_ FFU, the COGs target of ≤$3.50 per three dose course was met. At a clinical dose of 6.5 log_10_ FFU, the final manufacturing process resulted in a COGs that is substantially lower than the current average market price, 2.44 USD per dose. The manufacturing and formulation processes were transferred to BioFarma in Indonesia to enable future RV3-BB vaccine production.

## Highlights

• A low-cost manufacturing process for a neonatal rotavirus vaccine was developed.
• The formulation process developed resulted in a stable liquid vaccine at 2–8 °C.
• No pretreatment of vaccinees with antacid needed before oral administration.
• Tech. transfer package includes manufacturing, formulation, assays and COGs model.

## 1 Introduction

Rotavirus is a leading cause of mortality for neonates and children, in the absence of an effective vaccine nearly all children worldwide acquire a rotavirus infection by age five [1]. Symptoms include fever, vomiting and watery diarrhea which may lead to fatal dehydration [1]. Two rotavirus vaccines, RotaTeq® (Merck & Co, Kenilworth, NJ, USA) and Rotarix® (GlaxoSmithKline Biologicals, London, UK) were prequalified by the World Health Organization (WHO) in 2008 and 2009, respectively [2]. These vaccines have significantly reduced child mortality from gastroenteritis [1], [3], [4]. Despite availability of these vaccines, rotavirus remains one of the main causes of mortality among children under five years of age, with the majority of fatalities occurring in resource-poor settings in low-to-middle income countries with limited health care infrastructure and access [4], [5], [6]. Despite evidence of the success of rotavirus vaccines, more than 88 million infants still lack access to a rotavirus vaccine [6], [7]. Barriers to global implementation of the vaccine include cost, limited manufacturing capacity, suboptimal efficacy in low-income countries and safety concerns [5]. The 2018 WHO prequalification of ROTAVAC® (Bharat Biotech, Hyderabad, India) and ROTASIIL® (Serum Institute of India, Pune, India), and the availability of locally produced and licensed vaccines (Rotavin, Polyvac, Vietnam and Lamb rotavirus, Lanzhou Institute of Biological Products, China), should partially alleviate cost and supply barriers, however, there remains the challenge of sub-optimal efficacy of the current vaccines in low-income countries [4], [8]. Thus far, all licensed oral rotavirus vaccines had a high efficacy (>80%) in high- and middle income settings and lower efficacy (40–67%) in low-income settings despite having high vaccination coverage [4], [5]. Reasons for the lower efficacy remain unclear and interventions to improve efficacy in developing settings (e.g. withholding breastfeeding, adding buffers, micronutrient supplementation) have failed to yield definitive or actionable results [4], [5]. In addition, availability of a range of rotavirus vaccine options with heterogeneous characteristics including different presentations, dosing schedules, and prices can also present decision makers with more complex choices in selecting a vaccine product [9]. For example, dosages are indicated in different units such as cell culture infectious dose 50% (CCID50) vs FFU vs international units (IU), used in different schemes (2 dose vs 3 dose), and single versus multiple strains per dose (Table 1). In current vaccination schedules, a three dose rotavirus vaccine (RotaTeq®, ROTAVAC® and ROTASIIL®) is administered at 6–8, 10–14 and 14–18 weeks of age, or in a two dose vaccine schedule (Rotarix®) at 6–8 and 10–14 weeks (ranges as recommended by WHO and advised by the manufacturers) [10], [11], [12]. Therefore, neonates remain at risk of being exposed to rotavirus in the period from birth until 6–8 weeks of age before the first vaccine dose is given [6], indicating the need for a vaccine that can be administered directly at birth [13]. This is of particular importance in low- and lower-middle income countries (LICs and LMICs), where access to vaccines is poor, there is earlier onset of rotavirus disease, and the burden of disease is significant [6], [13]. The currently approved vaccines, listed above, have not been licensed with a neonatal dose.

**Table 1:** Summary of WHO prequalified rotavirus vaccines.

Rotavirus vaccine	Virus titer per aggregate dose	Unit	Regimen	Number of strains per dose	Administrationat weeks of age	Refe-rence
RotaTeq®	<8.1*	log_10_ IU	3-dose	Five	8, 14 and 18	[12]
Rotarix®	6.0	log_10_ CCID50	2-dose	One	8 and 14	[49]
ROTAVAC®	5.0	log_10_ FFU	3-dose	One	8, 14 and 18	[50]
ROTASIIL®	≥6.3**	log_10_ FFU	3-dose	Five	8, 14 and 18	[42]

* A minimum of 2.0–2.8 × 10^6^ infectious units (IU) per individual reassortant dose, depending on the serotype, and not greater than 116 × 10^6^ IU per aggregate dose.

** Recalculated for 5 strains from ≥10^5.6^ FFU/serotype.

To address current barriers and challenges, several new rotavirus vaccines are in different stages of pre-clinical and clinical development [5], [8], [14], and include live-attenuated, oral rotavirus, and non-replicating (e.g. virus-like-particles, recombinant subunit, or inactivated rotavirus), parenterally delivered rotavirus vaccines. One of the new promising live-attenuated oral rotavirus vaccines concepts is based on the RV3 rotavirus strain (G3P[6]) isolated from a newborn in Melbourne (Australia), and is currently under development at Murdoch Children’s Research Institute (MCRI), Australia.

The RV3-BB human neonatal rotavirus vaccine was developed to provide protection from severe rotavirus disease from birth [15], [16]. The attenuated human rotavirus strain (G3P[6]) has been shown to be associated with asymptomatic infection in newborns and provide protection against severe rotavirus infection in the first 3 years of life [15], [16]. Due to an earlier peak age of disease in high-mortality settings, the neonatal schedule may provide an earlier protection when compared with the infant schedule [17]. In a recent randomized placebo-controlled efficacy study conducted in Central Java and Yogyakarta, Indonesia, three doses of RV3-BB in Indonesian infants resulted in 75% efficacy against severe rotavirus gastroenteritis in the first 18 months of life when administered in a neonatal schedule (at ages 0–5 days, 8 weeks, and 14 weeks) compared with 51% when administered in an infant schedule (ages 8, 14, and 18 weeks), suggesting that the birth dose might enhance protection [3], [6]. The aim of the Central Java and Yogyakarta study was to investigate vaccine efficacy against severe rotavirus disease in the first 18 months of life [6]. A secondary outcome was to assess for impact of coadministration with OPV vs IPV on immunogenicity of RV3-BB rotavirus vaccine and OPV [3]. The co-administration of OPV with RV3-BB rotavirus vaccine in a birth dose strategy did not reduce the immunogenicity of either vaccine. These findings support the use of a neonatal RV3-BB vaccine in the routine vaccination schedule [3]. Moreover, use of RV3-BB produced vaccine take irrespective of histo-blood group antigen (HBGA) status, and showed potential to provide an improved protection in settings where P[6] rotavirus is endemic [18]. Human genetic diversity has an effect on rotavirus infections susceptibility and vaccine take [19]. Innate resistance to viral infections can be attributed to mutations in genes involved in the immune response, or to the receptor/ligand. This resistance appears to be rotavirus genotype-dependent and is mainly mediated by HBGAs, which function as a receptor or attachment factors on gut epithelial surfaces [19].

The RV3-BB vaccine is shown to be safe and immunogenic, and is currently in clinical phase III development at BioFarma, Indonesia [8], [14], with the intention to have a birth dose vaccination schedule [3], [6]. Another unique feature is that RV3-BB contains the targets P[6] genotype that may offer an advantage in regions (such as Africa and Asia) where P[6] strains are commonly associated with severe disease in children, as the vaccine appears to bind to receptors irrespective of HBGA status [18], [20]. Given these promising characteristics, we set out to develop a scalable and low-cost process, that allows for the robust large-scale production of a liquid RV3-BB rotavirus vaccine. Three major goals were established for the study: *(i)* the developed process should allow scale-up to large-scale commercial production of the RV3-BB vaccine at a desired manufacturing COGs of a maximum of 3.50 USD per complete vaccination course (expected to be 3 doses per course), *(ii)* the trypsin used for drug substance manufacturing should be of an animal-component-free, non-porcine, origin for safety reasons and *(iii)* the developed liquid vaccine formulation should be stable for two years at 2–8 °C and does not require pre-neutralization of gastric acid upon administration (i.e. vaccine is stable during long-term storage and during passage through the stomach). In regards to these vaccine formulation goals, it is known that licensed rotavirus vaccines use buffering excipients to minimize vaccine virus inactivation due to acidic conditions in the stomach [21]. Unfortunately, use of a separate buffering diluent results in the undesirable need for separate transport and storage and oral administration steps. In addition, oral administration of relatively large volumes (1.5 to 2.5 mL) of reconstituted or ready-to-use vaccine to infants is less preferred [21], [22]. Immunization programs prefer oral vaccines in smaller volumes (e.g. 0.5 to 1.0 mL) that are ready-to-use and do not require reconstitution or administration of separate components, such as a diluent or a buffer, to minimize errors in administration. The formulation development of the RV3-BB vaccine candidate that meets these vaccine dosage form objectives has been described in detail elsewhere [23], [24], [25].

In summary, the following studies provide for a manufacturing process that has the potential to meet the target COGs of ≤ 3.50 USD per complete vaccination course of 3 doses. In addition, the liquid oral rotavirus vaccine (ORV) formulation, intended for neonatal use, is stabilized at refrigerator temperature (2–8 °C), and could protect the virus from inactivation in the gastric acid environment of the stomach. We believe further studies are warranted as this RV3-BB vaccine candidate has the potential to address the sub-optimal efficacy, supply, and price challenges currently encountered in the fight against rotavirus.

## 2 Results

### 2.1 Production of RV3-BB virus

**Fig. 1:**
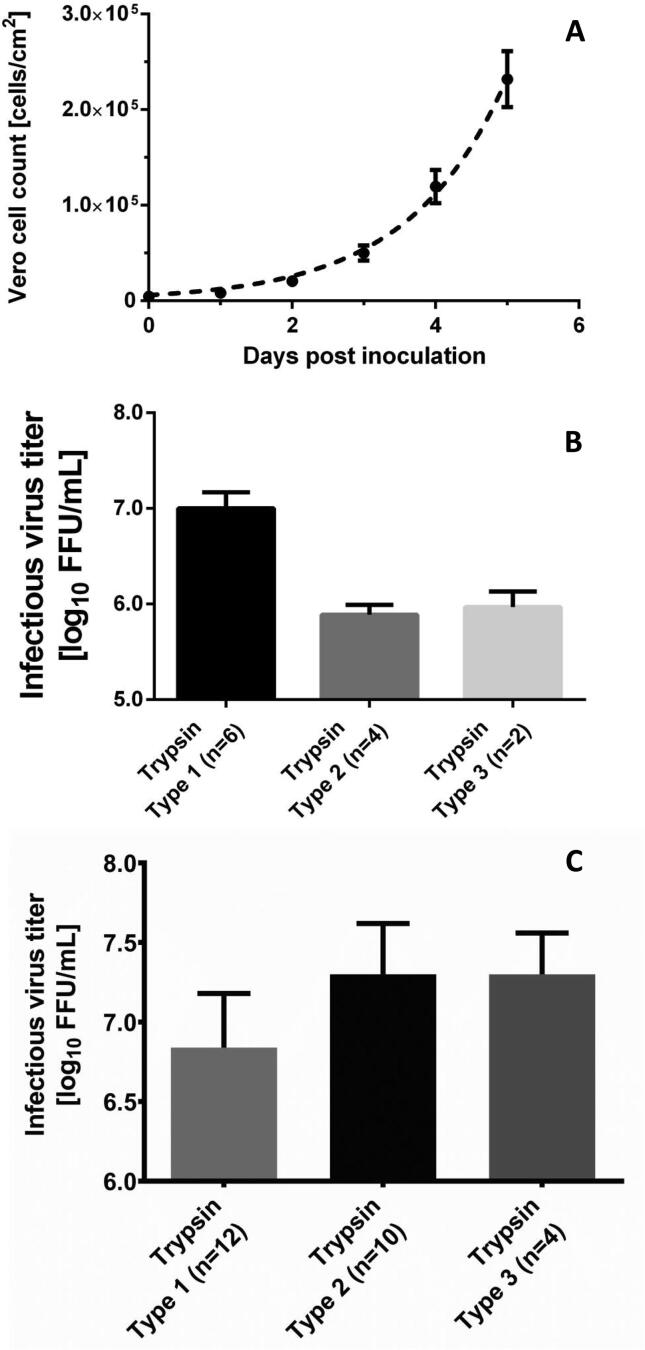
A) Reproducible Vero cell growth (n = 21; error bars indicate 95%CI) in the iCELLis® fixed-bed bioreactor. B) Rotavirus production in the iCELLis® fixed-bed bioreactor is dependent on the trypsin source used to activate the virus (error bars indicate standard deviation). C) Rotavirus production in Cell Factory systems. The same trypsin sources as used in the iCELLis® bioreactor (see figure B) show an opposite effect when used in the Cell Factory system (error bars indicate standard deviation; no significant difference was observed in the virus titers obtained between using trypsin type 2 compared with type 3). Cell Factory systems were selected as production system for RV3-BB. In the USP harvest, the obtained virus titer was on average 7.3 ± 0.3 log_10_ FFU/mL (n = 12), based on using Trypsin type 3.

Two cell culture systems were evaluated for manufacture of the rotavirus vaccine: an iCELLis® bioreactor and stacked cell culture flasks (Cell Factory™ system). Both systems are extensively used in the commercial vaccine and viral vector manufacturing industry [26], [27], [28], [29]. Based on the data shown in Fig. 1A it was concluded that the Vero cells could be readily cultured to 3.3 ± 0.7 × 10^5^ cells per cm^2^ (n = 21) in an iCELLis system, providing a high cell density biomass for the infection with the attenuated RV3-BB strain.

As little is known about the kinetics of rotavirus replication in Vero cells, and trypsin is pivotal for virus activation to facilitate cell entry, a number of studies were performed in Design of Experiments (DOE) mode in the iCELLis bioreactor. Parameters assessed included trypsin concentration at infection and in the maintenance medium, bed compaction, cell density at infection, multiplicity of infection, volume at infection, source of Vero cells, culture media feeding and harvest methods. From the data obtained (not shown) it was concluded that none of these parameters substantially improved RV3-BB virus titer in the iCELLis bioreactor with optimal results obtained in this system at 7.0 ± 0.2 log_10_ FFU/mL (n = 6), when using porcine trypsin.

One striking outcome from the DOE studies was related to use of trypsin in the two selected culture systems. As shown in Fig. 1B, trypsin type-1 (derived from porcine origin and used as reference) consistently provided the highest titer in the iCELLis bioreactor whereas trypsin type-2 and type-3 (both of animal-component-free origin) were superior in the Cell Factory system (Fig. 1C). Although poorly understood, one hypothesis is that the reduced accessibility of the cells in a fixed-bed bioreactor compared to a static cell culture system, combined with the chemical-physiological properties of the trypsin materials tested, significantly contributed to the difference seen in virus titer. Based on the results obtained, it was decided to continue the upstream cell and virus culture process development using the Cell Factory system. Typically, upon producing virus on a routine basis for purification studies using trypsin type-3, a virus titer of 7.3 ± 0.3 log_10_ FFU/mL (n = 12) was achieved in this system. In addition, the DOE studies performed in the Cell Factory system, enabled design of a production protocol that delivered the highest possible titer of RV3-BB virus including a substantial shortening of the process time (from overall 44 to 22 days) due to an adapted Vero cell preculture schedule, an optimal cell density at infection (CDAI) of 2.1 × 10^5^ cells/cm^2^, and a significant 10x reduction in the multiplicity of infection (MOI). All these findings were implemented in the cost modeling studies described below.

### 2.2 Purification of RV3-BB virus

**Fig. 2:**
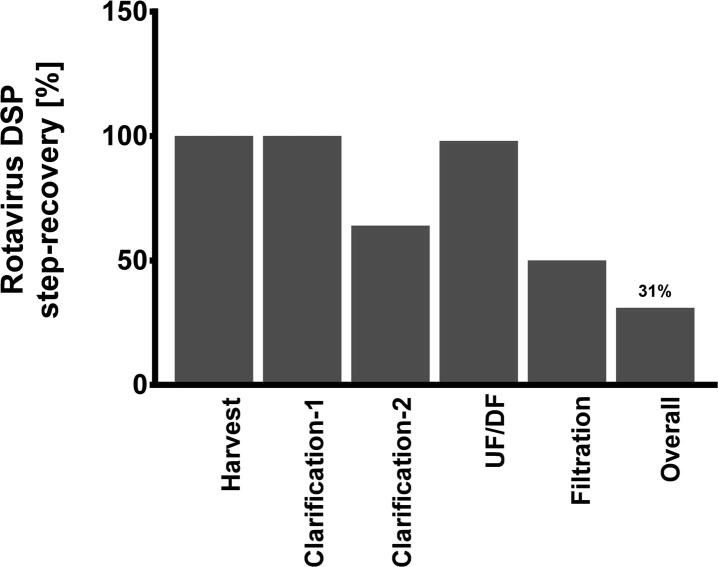
High infectious rotavirus recovery percentages per unit operation were obtained starting with the Cell Factory harvest, up to and including the UF/DF-stage. For aseptic processing, the cumulative virus recovery including the UF/DF-stage was >50%. However, the overall virus recovery was reduced to 31% when a filtration unit operation needs to be included.

**Fig. 3:**
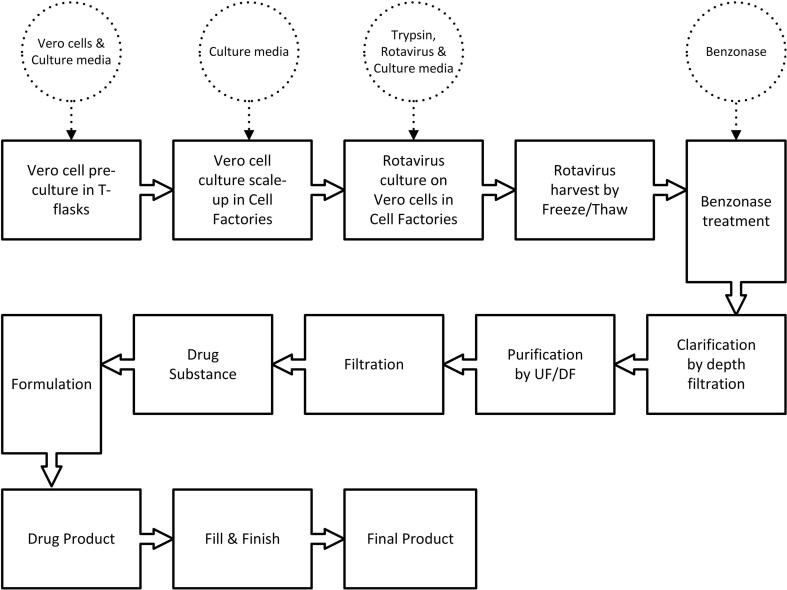
Process flow diagram for production of ORV. Solid lines indicate unit-operations; dashed circles indicate materials used.

Ultracentrifugation is a commonly used, lab-scale method for rotavirus purification. However, ultracentrifugation-based methods require expensive equipment, are challenging in scale-up, and less suitable for GMP manufacturing. Therefore, a scalable downstream process (DSP) was developed. Following virus harvest and virus release from the Vero cells by freeze-thawing (for Cell Factory harvests only), a DSP was developed consisting of (i) Benzonase® treatment, (ii) clarification, (iii) ultrafiltration/diafiltration (UF/DF) and (iv) a final filtration step. Overall RV3-BB virus recoveries obtained after each step are shown in Fig. 2. For development of a clarification step to reduce Vero host cell debris, a number of filter materials and pore sizes were screened resulting in a virus recovery of ≥80% at small scale (1–3 L volume) and >70% at larger scale (14 L volume). Prior to clarification, host-cell DNA digestion was performed using Benzonase®. After clarification, oligonucleotides were removed by ultrafiltration. During the UF/DF step, it was observed that an initial Benzonase® treatment was pivotal to reduce membrane fouling and improve virus recovery. The addition of Benzonase® resulted in a >1000-fold reduction of the host-cell DNA residuals (from 40,600 ng/ml to 20 ng/ml; Table 2). For live-attenuated vaccines such as rotavirus vaccines that are delivered orally, residual host-cell DNA should be limited to ≤100 μg/dose [30] [Ph. Eur. 10.0, 2417 (01/2012)], as orally administered DNA is absorbed approximately 10,000-fold less efficiently than parenterally administered DNA [31]. With the use of a final filtration step (0.2 µm pore-size), the overall recovery was reduced from >50% to 31% (Fig. 3). Although several different filter membranes, surfactants and buffers were tested, none improved the recovery of RV3-BB virus during this process step (data not shown). Here, it can be argued that the final filtration step could be omitted as a bioburden reduction step, in combination with aseptic processing, can suffice. Although this was not further modelled in these studies, omitting a final filtration step would clearly have a substantial impact on overall RV-3 BB virus recovery.

**Table 2:** In-process residuals levels following UF/DF, with and without the use of Benzonase® treatment prior to clarification.

In-process residual	With Benzonase® [ng/mL]	Without Benzonase® [ng/mL]
Host Cell DNA	20	40,600
BSA	<1	2
Host Cell Protein	5440	5557
Benzonase®	< LLOQ*	< LLOQ*

* LLOQ = Lower Level of Quantification = 5 ng/mL.

In summary and as shown schematically in Fig. 3, a drug substance (DS) manufacturing process was developed, delivering a yield of 7.3 ± 0.3 log_10_ FFU/mL in the upstream process (USP) utilizing Cell Factory systems and an overall RV3-BB virus recovery of ≥30% in the DSP post final filtration. An animal-component-free trypsin (type-3) was successfully introduced in the production process.

### 2.3 Analytical methods for RV-3 BB vaccine development

Assays described were developed according ICH Q2 analytical validation guidelines [32] and WHO guidelines [30]. To support release testing for bulk harvest, an identity polymerase chain reaction (PCR) was developed for virus identity testing and a fluorescent focus assay (FFA) was developed for the quantification of virus concentration. To support drug substance release testing, in addition to FFA, an assay was developed to quantify residual host-cell DNA. Additional tests were developed to support process development and validation and included residual bovine serum albumin (BSA), Benzonase® and host cell protein. An assay was not needed to assess the level of residual trypsin; a risk assessment determined that trypsin residuals were under the detection limit.

**Fig. 4:**
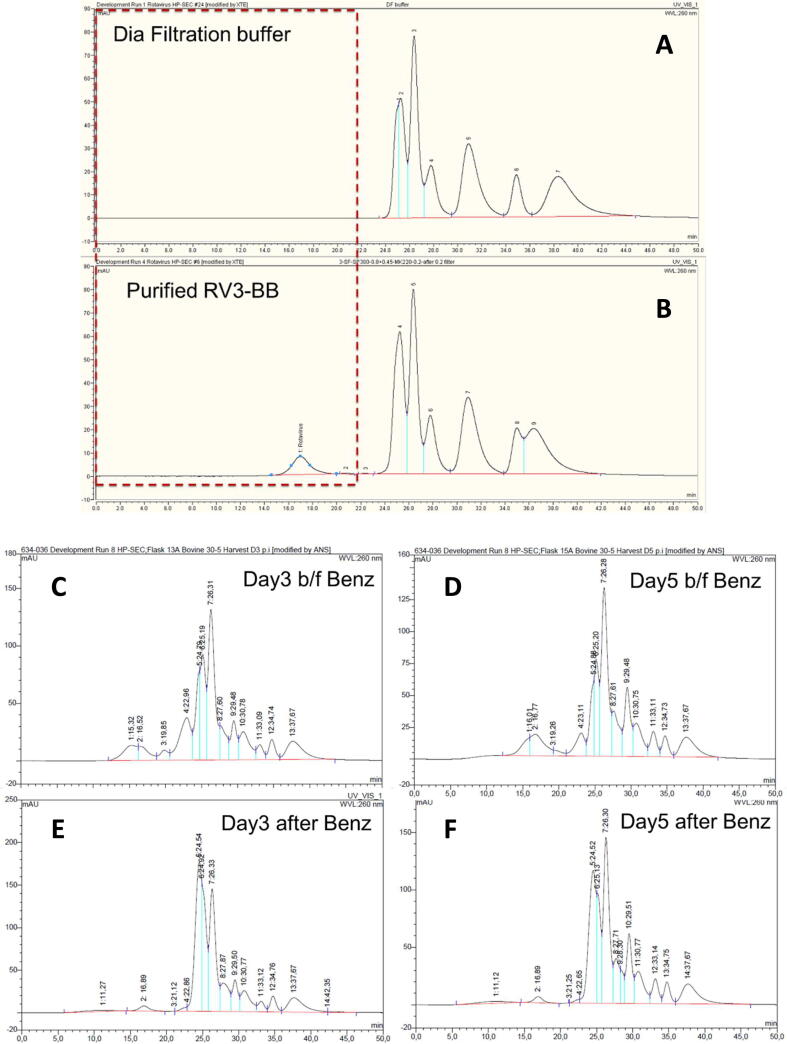
(A & B) HP-SEC analytical method for in-process rotavirus quantification to support process development. Specificity is demonstrated (A). At the retention time of rotavirus RV3-BB (around 17 min), no interfering peaks are observed for the diafiltration buffer. The purified rotavirus RV3-BB peak is baseline separated with resolution > 2 (B). Fig. 4 (C–F) The peak profile was observed to be shifting from day 1 to day 5 in USP process (day 3 and 5 are shown as examples in figure (C) and (D), which illustrates the rotavirus replication in USP. After Benzonase® treatment the profile shows a baseline separated peak at the retention time around 17 min (comparable to the purified RV3-BB peak; E and F). As a result, quantification of the rotavirus peak is possible after Benzonase® treatment.

In addition to the assays developed to support product release, a high-performance size-exclusion chromatography (HP-SEC) method for virus particle quantification was developed to support process development, process optimization and product characterization. The need for such an in-process assay became apparent while executing the DOE studies to optimize production of the RV-3 BB virus. Results of the novel HP-SEC method regarding assay specificity and quantification are shown in Fig. 4A and B, respectively. For total rotavirus particles analysis, the HP-SEC method was shown to be specific (baseline separated peak without matrix interference) and linearity was shown (r^2^ > 0.99) within a defined concentration range. Subsequently, the HP-SEC method was implemented in DSP for recovery calculations. During USP, the HP-SEC method was used for particle size profiling and in-process yield monitoring. In USP samples, virus quantification by HP-SEC was used following Benzonase® treatment of the sample. Based on the data obtained, it was concluded that this additional assay methodology (HP-SEC) for virus particle quantification delivered a semi-quantitative, high-throughput assay, without the relatively high variation, as is the case with biological assays such as the FFA. However, this method cannot differentiate between infectious and non-infectious viral particles. It is intended as an in-process method to quickly assess the virus yields. For product dosing the infectious titer needs to be determined separately.

### 2.4 Formulation of RV-3 BB virus

**Fig. 5:**
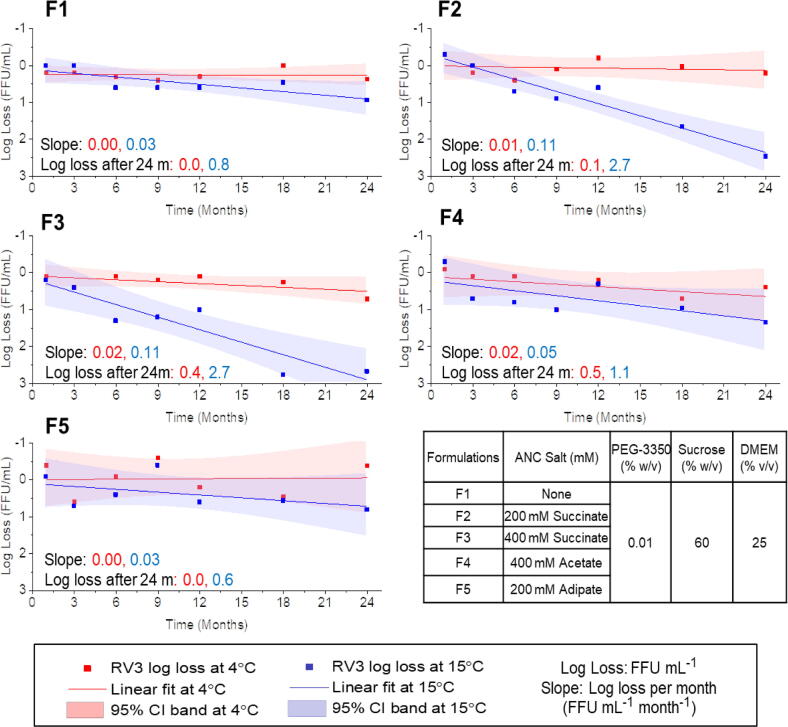
Storage stability profiles of RV3-BB in candidate liquid formulations (F1-F5) over 24 months at 2–8 °C and 15⁰C as measured by the FFA cell-based virus potency assay. The composition of the candidate formulations is shown in Table with each prepared in a phosphate buffer at pH 7.8. Solid lines (slope with units FFU mL^−1^ month^−1^) represent regression of mean log loss of RV3-BB viral titers at different temperatures and timepoints (squares) vs. –80⁰C control formulation run in the same FFA assay. Mean log loss values after 24 months based on slope values are also shown. Shaded areas represent 95% confidence interval of stability data.

As described in detail elsewhere [23], [24], [25], formulation development studies with RV3-BB identified a series of promising candidate liquid formulations. To investigate potentially stabilizing additives, ~50 different excipients from various excipient categories (e.g., sugars, salts, amino acids, polymers, buffering agents, etc.) were evaluated, and promising hits were identified. Then, different excipient combinations and concentrations were optimized. The selection of excipient “hits” was based on improvements in RV3-BB stability upon exposure to freeze–thaw, agitation and acidic pH conditions using an infectivity-qPCR potency assay as described elsewhere [23], [24]. A series of candidate RV3-BB liquid formulations were setup on accelerated and long-term stability studies and monitored using an infectivity-qPCR potency assay, with only selected samples being analyzed by the more labor intensive and time consuming FFA assay (i.e., the former being a higher-throughput assay used for formulation development and the latter used as the official potency assay). Accelerated and real-time stability data of a series of candidate RV3-BB formulations using the experimental infectivity qPCR assay are described elsewhere [23], [24]. In this work, two-year long-term RV3-BB stability data at 2–8 °C and 15 °C for some of the most promising candidate RV3-BB formulations (with varying levels of acid neutralizing capacity for oral administration without the need for pre-neutralization of gastric acid) as monitored by the official cell-based FFA potency assay are shown in Fig. 5. The selected RV3-BB candidate formulations displayed excellent stability profiles at 2–8 °C (with mean slope values of essentially no loss, 0.0 log_10_ FFU/mL) over 24 months in the absence of an acid neutralizing buffer (F1), and with values ranging 0.0 to 0.5 log_10_ FFU/mL total loss over 24 months in the presence of various amounts and types of acid neutralizing excipients (F2-F5). For example, the 2–8 °C RV3-BB stability profile after 24 months showed a trend of 0.0 log_10_ FFU/mL loss (with 200 mM adipic acid, F5), 0.1 log_10_ FFU/mL loss (with 200 mM sodium succinate, F2), 0.4 log_10_ FFU/mL loss (with 400 mM sodium succinate, F3) and 0.5 log_10_ FFU/mL loss (with 400 mM sodium acetate, F4). These log loss values are based on the mean slope values to facilitate comparisons of the various formulations, however, final shelf-life determination will be based on the lower 95% CI of the stability data to account for assay and process variability (see shaded area of Fig. 5). The candidate RV3-BB liquid formulations were more stable at 2–8 °C as compared to 15 °C, the latter temperature allowing for better differentiation between the candidate formulations (Fig. 5). In addition, these RV3-BB formulations did not show any viral infectivity losses when stored frozen at –20 °C for 24 months (data not shown). This promising real-time RV3-BB stability data over 24 months in candidate formulations support the implementation of a refrigerator stable, liquid formulation (see discussion).

### 2.5 RV-3 BB vaccine: Cost of goods analysis

BioSolve® software was used at Batavia Biosciences for COGs calculations; a Drug Substance Cost of Goods (COGs) model was developed, and validated in collaboration with BDO’s BioProcess Technology Group (BDO, Boston, MA, USA) and Duff & Phelps (D&P, New York, NY, USA) in accordance with methodology developed with the Bill & Melinda Gates Foundation [33] using SuperPro® software. Prior to analysis of the calculated manufacturing costs for the RV3-BB Oral Rotavirus vaccine (ORV), the cost model was validated using data on UNICEF tender prices for Global Alliance for Vaccines and Immunization (GAVI) LIC countries in the period 2014–2018 (based on data from WHO [34], assuming a marginal profit margin for this category of countries. In this period, ORV (Rotarix® by GlaxoSmithKline Biologicals SA, and RotaTeq® by Merck Vaccines) tender prices for GAVI LIC countries were on average 2.44 USD per dose, and ranged from 1.89 to 3.62 USD per dose [34]. Based on this information, the target COGs for manufacturing (i.e., not the anticipated sales price) of our RV3-BB vaccine was set below this range at 1.17 USD per dose (or 3.50 USD for three doses).

**Fig. 6:**
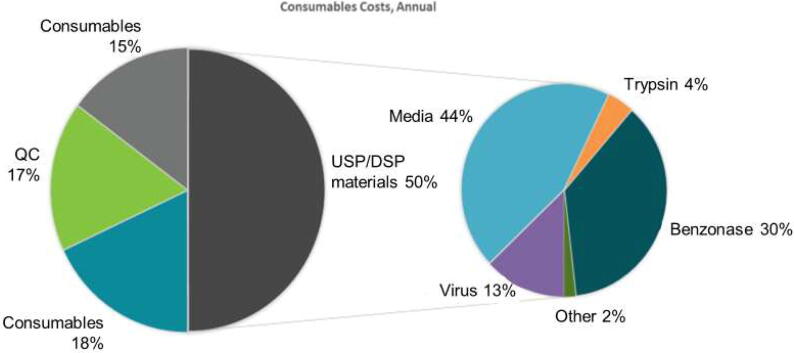
Main cost drivers were determined based on the COGs model. Benzonase® contributed 10% to the overall COGs. Alternatives for Benzonase® were identified based on the COGs calculations.

Details of the developed production process were entered into the software model (this excluded R&D costs, marketing and distribution costs, and profit margins). For the drug substance COGs model, the basic unit operations included 64 Cell Factory systems in one run with the harvests pooled to perform one DSP run. The COGS analysis showed that USP consumables accounted for 50% of the materials costs (Fig. 6). Among the USP consumables, both culture media and Benzonase® were identified as major cost drivers. Based on the process developed in this study, a COGs well below the target of ≤$3.50 per course can be delivered at the lower (6.0 log_10_ FFU) clinical dose tested in the dose ranging study. If the middle dose of 6.5 log_10_ FFU is determined as the clinical dose, the COGs will be approximately $1.40 above the target of $3.50, but still substantially below the current average market price of 2.44 USD per dose ($7.32 per three dose course; 2014–2018 UNICEF tender prices).

## 3 Discussion

A drug substance manufacturing process for RV3-BB neonatal rotavirus vaccine bulk production was developed and met the manufacturing COGs target of less than 3.50 USD per complete vaccination course of three doses. The novel virus production process was designed such that it allows 16 runs on a yearly basis. In virus production, the multiplicity of infection (MOI) was optimized for 10-fold more efficient use of master virus seed-stocks, thus lowering the COGs. The process consistently delivered a harvest with the rotavirus titer at 7.3 ± 0.3 log_10_ FFU/mL and incorporated use of an animal-origin free, GMP-grade, source of trypsin. For virus purification, a robust and scalable 4-step process was developed providing overall DSP infectious rotavirus recovery of approximately 30% (including final filtration), or alternatively >50% when excluding final filtration (requiring aseptic processing). The relatively high loss of infectious virus observed with final filtration can best be explained by virus clogging the filter pores, seeing that the virus diameter (77 nm) and the pore size (200 nm) are in the same order of magnitude. Host-cell DNA impurity level in drug substance and calculated in drug product were significantly (>99.9%) below WHO and European Pharmacopoeia human oral rotavirus vaccine regulatory requirements. Finally, a novel HP-SEC analytical method for virus particle quantification was developed, which can be used to support future fast-track process development, delivering a semi-quantitative high throughput methodology, without the relatively high assay variation as observed with biological assays such as FFA.

This rotavirus vaccine process is expected to meet the targeted low COGs needed for new vaccine manufacturers to implement the technology, build a sustainable business case, and compete with currently available rotavirus vaccines. The suitability of a lower titer vaccine (6.0, 6.5 and 7.0 Log_10_ FFU) is currently under clinical investigation in a dose ranging study performed in Malawi by MCRI (ClinicalTrials.gov Identifier: NCT03483116) and may contribute to efforts to lower the vaccine cost per dose [35]. In addition, further process optimization opportunities were identified in USP (use of fixed-bed bioreactors in combination with trypsin alternatives) and DSP (use of lower cost alternatives for Benzonase® (e.g., DNArase, which could further reduce overall COGs by 10%), use of aseptic processing, or alternative final filtration methods to increase the overall virus recovery), and may contribute to further reduction of COGs.

In addition, candidate RV3-BB formulations resulting in a 2–8 °C stable liquid vaccine which do not require pre-neutralization of gastric acid prior to administration have been developed (as described in detail elsewhere [23], [24]). In this work, 2-year, real-time (2–8 °C) and accelerated (15 °C) stability data with some of the key candidate RV3-BB liquid formulations as measured by the FFA viral infectivity assay are presented. The development of a stable liquid formulation of a live, attenuated viral vaccine can be challenging due to the inherent instability of live viruses and variability observed in viral infectivity assays [36]. Moreover, additional factors including the “stability window” between the viral titer required at release (highest dose that is safe) and expiry (lowest dose that is efficacious) for a specific vaccine candidate must be considered as part of process development and clinical trials [37]. Because of the observed promising RV3-BB real time storage stability profiles demonstrated in this work (Fig. 5), a lower virus concentration in the vial can potentially now be targeted (depending on final selection of target dose based on ongoing Malawi clinical trials; see above), which in turn could contribute to lowering the overall COGs. Although a stable liquid RV3-BB liquid formulation is expected based on this work, final determination of shelf-life and VVM designation will require future determination of (1) storage stability profiles of RV3-BB bulks in the selected final formulation produced in the final manufacturing facility and filled into the commercial primary container, (2) determination of lower 95% CI of the stability data assayed by the final version of the FFA cell based potency assay, and (3) the clinically required RV3-BB virus dose at release and expiry. In addition, accelerated stability data using the FFA assay will need to be obtained with RV3-BB virus from the final manufacturing conditions as part of future work. These data will be used to determine the Vaccine Vial Monitor (VVM) designation of the RV3-BB vaccine candidate in the final formulation [38]. Currently, initial short-term accelerated stability data are being collected for some of these candidate RV3-BB liquid formulations using an experimental viral infectivity qPCR assay, and assessments of the ability to model accelerated stability data (15, 25 and 37 °C) to predict long-term, real-time stability data (2–8 °C) are ongoing and will be described separately [25].

We thus describe a robust production protocol for a 2–8 °C stable liquid formulation of the RV3-BB virus vaccine candidate. Historically, when using FRhL-2 cells in rotavirus vaccine manufacturing, reported virus titers were approximately 10^5^ – 10^8^ PFU/mL, and purification was not required [39]. Thus the vaccine dose of 10^5^ PFU/re-assortant/dose could simply be obtained by dilution [39]. Currently, the licensed rotavirus vaccines (RotaTeq®, Rotarix®, ROTAVAC® and ROTASIIL®) are Vero cell-derived [40], [41], [42]. However, no peer reviewed details have been published regarding the applied vaccine manufacturing processes, cell and virus culture methods, purification, and filtration methods or the observed in-process yields. We therefore embarked upon our studies with an openness to consider a wide range of possible options for the process being developed.

In the upstream process developed for RV3-BB, several advantages were offered by use of the iCELLis® bioreactor for RV3-BB production. These included a small manufacturing footprint, large bed size of 500 m^2^ for scale-up, relative ease of use, and as a result, the potential for a low COGs if acceptable yields could be obtained. Yield comparison between iCELLis® nano (6.0 ± 0.2 log_10_ FFU/mL) and Cell Factory systems (7.3 ± 0.3 log_10_ FFU/mL), however, showed that titers obtained in the Cell Factory systems were in the expected and required range, when using a non-animal trypsin source, and thus this cell culture system was selected to continue for further process development.

To increase virus production yields through process optimization, three key factors were considered: i) cell concentration and metabolic/physiological status of the cells at time of infection, ii) ratio of infectious particles to viable cells at the time of infection, and iii) residence time of virus particles within the bioreactor and time point of harvest [43]. Regarding the residence time, once maximum titers have been achieved, virus infectivity and the total number of virus particles can decrease again [39]. Based on this knowledge, optimization strategies were introduced and included shortening the process time due to an adapted Vero cell preculture schedule (for increased yearly production capacity) and screening of process parameters as cell density at infection (CDAI), MOI and cell growth and infection media. Ultimately, the process was fixed using a MOI 10x lower compared to the initial process to make efficient use of master virus seed (MVS) stocks.

When using diploid host cells such as FRhL-2 cells, moderate to no purification is required for rotavirus vaccine due to the oral delivery and the cell substrate used [39], [44]. With the use of other cell lines such as Vero, for example, alternative rotavirus purification approaches using chromatographic methods have been proposed in the literature [45], [46] as well as alternative membrane chromatography purification methods for rotavirus-like particles [47], [48]. In follow-up studies, these alternative methods could be considered in the DSP to achieve comparable impurity removal and improved recovery. Alternatively, omitting the final sterile filtration step in the process developed here for RV3-BB will deliver a significantly increased DSP recovery and can be used in combination with an aseptic process for the manufacturing of this oral vaccine.

**Fig. 7:**
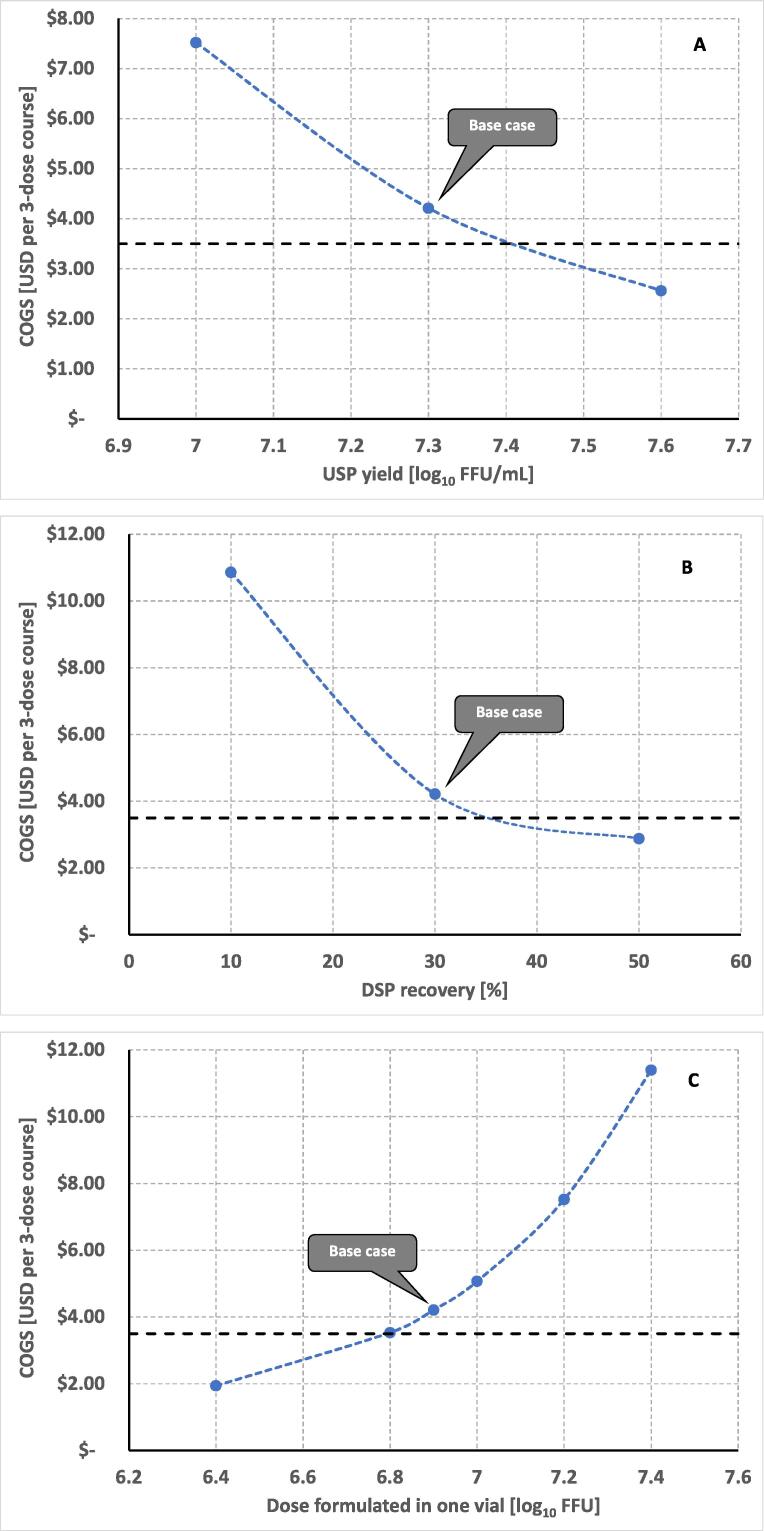
(A-C) USP Yield (Fig. 7A), DSP recovery(Fig. 7B) and vial fill dose (Fig. 7C) scenario analyses. Base cases indicate the applicable base-case process (harvest yield 7.3 log_10_ FFU/mL and DSP recovery of 30%) and formulated dose (6.9 log_10_ FFU in Phase I clinical trial). Dashed line indicates opportunities to reach the targeted cost (USD 3.50) per three dose course.

The Cell Factory system was chosen for the current process development, and for the COGs scenario, a comparable overall facility capacity between 34 and 40 M doses per annum was assumed. The overall drug product COGs range estimation for both USP system alternatives (i.e. iCELLis® fixed-bed bioreactor versus Cell Factory systems) appeared comparable. This overall DP COGs range was independently confirmed using alternative software (SuperPro Designer®). Main cost drivers were culture media and Benzonase® for both USP options, followed by trypsin when using the iCELLis® system; the cost of Benzonase® overtaken by labor for the Cell Factory-based process. Using the developed COG’s model, scenario analyses were performed by an external independent party (BPTC/D&P), and included one, five, and ten dose vial fills (Fig. 7). If, for example, the cost objective was fixed at the indicated future large-scale lower COGs target level of 3.50 USD per course (expected to consist of three doses per course and filled at five doses per vial), this goal can be achieved in only a limited number of scenarios: (i) an increased USP virus titer, and/or (ii) an increased overall DSP recovery level, and/or (iii) a decreased clinical dose level.

While scenario i) was feasible using the currently developed process, scenarios ii) and iii) could be explored further to achieve the lower cost objective. In DSP, selection of alternative final filtration methods, or the application of aseptic processing, may increase overall DSP recovery significantly (>50%), resulting in achievement of an even lower cost per course objective. Alternatively, the cost target may be further reduced by choosing a lower dose.

Recently, the direct vaccine cost (excluding waste) for GAVI-supported rotavirus vaccines in the period 2019–2021 was reported to range lower, between 0.85 and 2.29 USD per dose [10]. One dose of these vaccines contain 6.0 log_10_ CCID50 (used in a 2-dose regimen) per dose, 5.0 log_10_ FFU (used in a 3-dose regimen) per dose, and ≥5.6 log_10_ FFU for five strains each per dose (used in a 3-dose regimen), for Rotarix® [49], ROTAVAC® [50], and ROTASIIL® [42], respectively. Feasibility of this COGs range is also illustrated by the recently introduced and WHO prequalified rotavirus vaccine (ROTAVAC®) by Bharat Biotech. ROTAVAC® was initially reported to be offered at 0.95 USD/dose to the Government of India [51], and currently priced (for the period 2017–2021) at 0.85 USD/dose for UNICEF [52]. To enable this even lower direct RV3-BB vaccine cost, the main cost drivers and several opportunities for process optimization were identified above. From the COGs data, it was observed that with high yearly facility capacity (>40 M doses/year), use of a fixed-bed bioreactor could lower the cost per dose when using a certain trypsin source.

The lowest and middle dose both resulted in the COGs still substantially below the current average market price of 2.44 USD per dose ($7.32 per three dose course; 2014–2018 UNICEF tender prices). The COGs of the lowest clinical dose were well below the target of ≤$3.50 per course. For the middle dose the COGs will be approximately $1.40 above the target of $3.50, but still substantially below the current average UNICEF tender price. In addition, we have identified several areas by which the process COGs can be further reduced, for example by replacing the Benzonase® with a lower cost alternative such as DNArase, which could reduce COGs by 10%. Further, the above mentioned COGs is for a process including a final sterile filtration step, a process step that can be omitted when producing an Oral vaccine under aseptic conditions.

## 4 Materials & methods

Cell line: WHO Vero 10–87 derived working cell banks were used. Initially, the Batavia Vero working cell bank was used for small scale screening experiments. During drug substance manufacturing process development, the BioFarma Vero working cell bank was used for process confirmation and local implementation.

Virus: The RV3 strain was isolated at the Royal Children's Hospital (Melbourne, Australia), and currently further developed as the RV3-BB vaccine at Murdoch Children’s Research Institute [6], [15], [16]. Clinical trial lots for phase I, IIa, IIb and dose ranging studies conducted by MCRI were manufactured under GMP by Meridian Life Sciences, Memphis (USA) at a titer of 8.6 × 10^6^ FU/ml. RV3-BB vaccine was provided to BioFarma to manufacture the RV3-BB under license from MCRI. A vial of the BioFarma RV3-BB working virus bank was passaged four times on Vero cells to generate a research virus bank used for the experiments.

Culture media and chemicals: Commercial cell and virus culture media, Bovine Serum from certified TSE free sources was used in USP, trypsin, and animal-component-free trypsin alternatives were used. Benzonase® was obtained from Merck Chemicals.

Cell culture systems: T-flasks (Greiner), iCELLis® nano (fixed-bed bioreactor; PALL), and scale-X Hydro (fixed-bed bioreactor; Univercells) were used in the cell culture system selection stage. In the drug substance manufacturing process development stage, Cell Factory systems (Easy Fill CF10; Thermo Fisher Scientific) were used.

Analytical methods: ELISA methods were applied for Vero host-cell protein (Cygnus Technologies, catalog nr. F500), residual BSA (Cygnus Technologies, catalog nr. F030), and residual Benzonase® (Merck, catalog nr. 1.01681.0001). For Vero host-cell DNA, a QPCR method was applied (Thermo Fisher, catalog nr. 4460367). The Focus Forming Assay (FFA) was developed as a content/potency assay according to ICH Q2 guidelines [32]. In the FFA, MA104 cells are infected with rotavirus and the read-out is by fluorescence microscopy to count and quantify the number of fluorescent cells. Results of the FFA are expressed as focus forming units per milliliter, or FFU/mL.

Cost modelling (software and assumptions made): Based on the process development at Batavia, and using industrial manufacturing costs (including depreciation time and rate, exchange rates, facility availability, overhead, maintenance, implementation of single-use bioreactor and buffer preparation systems, waste management, working hours, personnel, materials, packaging, licensing, and distribution) input from BioFarma, a drug substance COGs model was developed, and validated in collaboration with BDO’s BioProcess Technology Group (BDO, Boston, MA, USA) and Duff & Phelps (D&P, New York, NY, USA). The initial drug substance COGs model was developed using BioSolve Process software (v7) (Biopharm Services Ltd., Chesham, UK), and the model was validated independently using SuperPro Designer® (v9) (Intelligen Inc, Scotch Plains, NJ, USA) by BDO’s BioProcess Technology Group. In addition, a fill and finish model were developed in SuperPro Designer®, to also include drug product cost estimations.

The cost models were defined by a detailed process description including USP and DSP unit operations, process scale (equipment sizing), product titers and resources allocation. A cost database, which is built with data consisting of benchmarking information including equipment and materials, is coupled to calculate costs for the process. Together with the required utilities, the manufacturing COGs can be determined. The complete ORV manufacturing process (3) was described to calculate the manufacturing costs per dose for two process options, based on Cell Factory™ or iCELLis® use in USP, to assess the cost drivers, and identify targets for the reduction of the COGs.

Formulation and stability: The development and preparation of candidate RV3-BB formulations, along with the design of the accelerated and real-time stability program, is described in detail elsewhere [23]. Briefly, selected candidate formulations were prepared, mixed with RV3-BB virus bulks, filled into stoppered glass vials. Samples were removed at indicated times and temperatures, stored at −80 °C and subsequently assayed for RV3 infectivity values by FFA assay. At each stability time point, the samples stored at 2–8 °C and 15 °C were assayed along with the same candidate formulation stored frozen at −80 °C. Stability values are expressed as log loss vs the −80 °C control formulation. This approach improves stability estimations by lowering assay variability as described in detail elsewhere [25].

## Declaration of Competing Interest

The authors declare the following financial interests/personal relationships which may be considered as potential competing interests: JEB is the program lead of the RV3 Rotavirus Vaccine Program at Murdoch Children’s Research Institute that is aiming to license the RV3-BB vaccine.

## References

[1] Clark A. (2017). Estimating global, regional and national rotavirus deaths in children aged <5 years: Current approaches, new analyses and proposed improvements. PLoS ONE.

[2] GAVI-supported rotavirus vaccines profiles to support country decision making - rotavirus-vaccine-profilespdf.pdf. 2019 [cited 2020 April 02]; Available from: https://www.gavi.org/our-alliance/market-shaping/product-information-vaccines-cold-chain-equipment.

[3] Cowley D. (2019). Immunogenicity of four doses of oral poliovirus vaccine when co-administered with the human neonatal rotavirus vaccine (RV3-BB). Vaccine.

[4] Steele A.D. (2019). Experiences with rotavirus vaccines: can we improve rotavirus vaccine impact in developing countries?. Hum Vaccin Immunother.

[5] Jiang B., Patel M., Glass R.I. (2019). Polio endgame: Lessons for the global rotavirus vaccination program. Vaccine.

[6] Bines J.E. (2018). Human neonatal rotavirus vaccine (RV3-BB) to target rotavirus from birth. N Engl J Med.

[7] International Vaccine Access Center (IVAC), Johns Hopkins Bloomberg School of Public Health. VIEW-hub. www.view-hub.org. 2020 [cited 2020 24 September]; Available from: https://view-hub.org/map/?set=children-without-access&group=vaccine-access&category=rv.

[8] Burke R.M. (2019). Current and new rotavirus vaccines. Curr Opin Infect Dis.

[9] Pecenka C. (2020). Cost-effectiveness analysis for rotavirus vaccine decision-making: How can we best inform evolving and complex choices in vaccine product selection?. Vaccine.

[10] GAVI - Vaccine Detailed Product Profiles. 2020 [cited 2020 March 26]; Available from: https://www.gavi.org/news/document-library/detailed-product-profiles.

[11] Summary of WHO Position Papers - Recommended Routine Immunizations for Children. 2019 [cited 2020 August 26]; Available from: https://www.who.int/immunization/policy/Immunization_routine_table2.pdf?ua=1.

[12] Rotateq product insert - Highlights of prescribing information. 2018 [cited 2020 July 01]; Available from: https://www.merck.com/product/usa/pi_circulars/r/rotateq/rotateq_pi.pdf.

[13] Clark A. (2019). Mortality reduction benefits and intussusception risks of rotavirus vaccination in 135 low-income and middle-income countries: a modelling analysis of current and alternative schedules. Lancet Glob Health.

[14] Kirkwood C.D. (2019). The rotavirus vaccine development pipeline. Vaccine.

[15] Danchin M. (2013). Phase I trial of RV3-BB rotavirus vaccine: a human neonatal rotavirus vaccine. Vaccine.

[16] Bines J.E. (2015). Safety and immunogenicity of RV3-BB human neonatal rotavirus vaccine administered at birth or in infancy: a randomised, double-blind, placebo-controlled trial. Lancet Infect Dis.

[17] Clark A. (2019). Efficacy of live oral rotavirus vaccines by duration of follow-up: a meta-regression of randomised controlled trials. Lancet Infect Dis.

[18] Boniface K. (2020). Human neonatal rotavirus vaccine (RV3-BB) produces vaccine take irrespective of histo-blood group antigen status. J Infect Dis.

[19] Sharma S. (2020). The impact of human genetic polymorphisms on rotavirus susceptibility, epidemiology, and vaccine take. Viruses.

[20] Cowley D. (2018). Molecular characterisation of rotavirus strains detected during a clinical trial of the human neonatal rotavirus vaccine (RV3-BB) in Indonesia. Vaccine.

[21] Ella R. (2018). A Phase 4, multicentre, randomized, single-blind clinical trial to evaluate the immunogenicity of the live, attenuated, oral rotavirus vaccine (116E), ROTAVAC(R), administered simultaneously with or without the buffering agent in healthy infants in India. Hum Vaccin Immunother.

[22] Mansoor O.D. (2013). Vaccine Presentation and Packaging Advisory Group: a forum for reaching consensus on vaccine product attributes. Bull World Health Organ.

[23] Kumar P, Shukla RS, Patel A, Pullagurla SR, Bird C, Oluwadara Ogun, et al. Formulation development of a live attenuated human rotavirus (RV3-BB) vaccine candidate for use in low and middle-income countries; 2020 [submitted for publication]. 10.1080/21645515.2021.1885279 PMC8189091 33861183

[24] Kumar P, Pullagurla SR, Patel A, Shukla RS, Bird C, Kumru OS, et al. Effect of formulation variables on the stability of a live, rotavirus (RV3-BB) vaccine candidate using in vitro gastric digestion models to mimic oral delivery. J Pharm Sci; 2020 [in press]. 10.1016/j.xphs.2020.09.047 PMC7815322 33035539

[25] Pullagurla SR, Kumar P, Ogun O, Kumru OS, Hamidi A, Hoeksema F, Yallop C, et al. Modeling the long-term stability profiles of a live, rotavirus (RV3-BB) vaccine candidate in various liquid formulations via extrapolations of real-time and accelerated stability data; 2020 [in preparation]. 10.1016/j.biologicals.2021.12.001 34924260

[26] Leinonen H.M. (2019). Preclinical Proof-of-concept, analytical development, and commercial scale production of lentiviral vector in adherent cells. Mol Ther Methods Clin Dev.

[27] Rajendran R. (2014). Assessment of packed bed bioreactor systems in the production of viral vaccines. AMB Express.

[28] Valkama A.J. (2018). Optimization of lentiviral vector production for scale-up in fixed-bed bioreactor. Gene Ther.

[29] Lesch H.P. (2015). Process development of adenoviral vector production in fixed bed bioreactor: from bench to commercial scale. Hum Gene Ther.

[30] WHO TRS 941 Annex 3 - Guidelines to assure the quality, safety and efficacy of live attenuated rotavirus vaccines (oral). 2007 [cited 2020 April, 03]; Available from: https://www.who.int/biologicals/publications/trs/areas/vaccines/rotavirus/en/.

[31] Vernay O. (2019). Comparative analysis of the performance of residual host cell DNA assays for viral vaccines produced in Vero cells. J Virol Methods.

[32] ICH Q2 Analytical Validation - Q2(1) Guideline.pdf. 2005 [cited 2020 April 02]; Available from: https://www.ich.org/page/quality-guidelines.

[33] BMGF - Production Economics Vaccines. 2016 [cited 2020 August 26]; Available from: https://docs.gatesfoundation.org/Documents/Production_Economics_Vaccines_2016.pdf.

[34] WHO - Market Information for Access to Vaccines - MI4A Vaccine Purchase Data - MI4A/V3P vaccine purchase database. 2020 [cited 2020 April 02]; Available from: https://www.who.int/immunization/programmes_systems/procurement/mi4a/platform/module1/en/.

[35] A Phase II Dose-ranging Study of Oral RV3-BB Rotavirus Vaccine. 2020 [cited 2020 April 02]; Available from: https://clinicaltrials.gov/ct2/show/NCT03483116.

[36] Kumru O.S. (2014). Vaccine instability in the cold chain: mechanisms, analysis and formulation strategies. Biologicals.

[37] Egan W, Schofield T. Basic principles of stability. Biologicals 2009;37(6): 379–86; discussion 421–3. 10.1016/j.biologicals.2009.08.012 19720547

[38] Vaccine vial monitor (VVM) assignments for different WHO-prequalified vaccines and their proper handling. [cited 2020 August 28]; Available from: https://www.who.int/immunization/programmes_systems/service_delivery/EN_Information_Bulletin_VVM_assignments.pdf?ua=1.

[39] Auniņš JG. Viral vaccine production in cell culture. Encyclopedia of Industrial Biotechnology: Bioprocess, Bioseparation, and Cell Technology; 2009. p. 1–35.

[40] Barrett P.N. (2009). Vero cell platform in vaccine production: moving towards cell culture-based viral vaccines. Expert Rev Vaccines.

[41] Bhandari N. (2009). A dose-escalation safety and immunogenicity study of live attenuated oral rotavirus vaccine 116E in infants: a randomized, double-blind, placebo-controlled trial. J Infect Dis.

[42] Kulkarni P.S. (2017). A randomized Phase III clinical trial to assess the efficacy of a bovine-human reassortant pentavalent rotavirus vaccine in Indian infants. Vaccine.

[43] Tapia F. (2016). Bioreactors for high cell density and continuous multi-stage cultivations: options for process intensification in cell culture-based viral vaccine production. Appl Microbiol Biotechnol.

[44] Aunins J.G. (2011). Chemical engineering perspectives on vaccine production. Chem Eng Prog.

[45] Yang X. (2012). Purification of rotavirus by two chromatographic methods. Chinese J Biol.

[46] Farkas K. (2013). A gel filtration-based method for the purification of infectious rotavirus particles for environmental research applications. Food Environ Virol.

[47] Peixoto C. (2007). Downstream processing of triple layered rotavirus like particles. J Biotechnol.

[48] Vicente T. (2008). Anion-exchange membrane chromatography for purification of rotavirus-like particles. J Membr Sci.

[49] Rotavirus vaccines. WHO position paper - January 2013. Wkly Epidemiol Rec 2013;88(5): 49–64. 23424730

[50] Chandola T.R. (2017). ROTAVAC((R)) does not interfere with the immune response to childhood vaccines in Indian infants: a randomized placebo controlled trial. Heliyon.

[51] Lopez A.L., Raguindin P.F., Silva M.W.T. (2019). Prospects for rotavirus vaccine introduction in the Philippines: Bridging the available evidence into immunization policy. Hum Vaccin Immunother.

[52] UNICEF Supplies and Logistics - Rotavirus Vaccines (RV) - Rotavirus Vaccine Supply & Demand Update - RV_5_Supply_Updated.pdf. 2020 [cited 2020 April 02]; Available from: https://www.unicef.org/supply/index_70173.html.

